# A refined information processing capacity metric allows an in-depth analysis of memory and nonlinearity trade-offs in neurocomputational systems

**DOI:** 10.1038/s41598-023-37604-0

**Published:** 2023-06-29

**Authors:** Tobias Schulte to Brinke, Michael Dick, Renato Duarte, Abigail Morrison

**Affiliations:** 1 Institute of Neuroscience and Medicine (INM-6) and Institute for Advanced Simulation (IAS-6) and JARA-BRAIN Institute I, Jülich Research Centre, 52425 Jülich, Germany; 2 Department of Computer Science 3 - Software Engineering, RWTH Aachen University, Aachen, Germany; 3 Peter Grünberg Institut (PGI-1) and Institute for Advanced Simulation (IAS-1), Jülich Research Centre, 52425 Jülich, Germany; 4 Donders Institute for Brain, Cognition and Behavior, Radboud University, Nijmegen, The Netherlands

**Keywords:** Dynamical systems, Dynamical systems

## Abstract

Since dynamical systems are an integral part of many scientific domains and can be inherently computational, analyses that reveal in detail the functions they compute can provide the basis for far-reaching advances in various disciplines. One metric that enables such analysis is the information processing capacity. This method not only provides us with information about the complexity of a system’s computations in an interpretable form, but also indicates its different processing modes with different requirements on memory and nonlinearity. In this paper, we provide a guideline for adapting the application of this metric to continuous-time systems in general and spiking neural networks in particular. We investigate ways to operate the networks deterministically to prevent the negative effects of randomness on their capacity. Finally, we present a method to remove the restriction to linearly encoded input signals. This allows the separate analysis of components within complex systems, such as areas within large brain models, without the need to adapt their naturally occurring inputs.

## Introduction

Dynamical systems are systems whose state evolves over time, following specific laws of motion. They are often defined by differential equations that characterize the motion of their elements in a finite state-space^1^. The theory of such systems originates in the study of the motions of celestial bodies^2^, but today finds applications in many research fields. From the double pendulum^3^, to the modelling of population dynamics^4^ and the interacting reactions of chemicals^5^, to fields such as the study of collective decision-making processes^6^, infant development^7^ or brain development^8^, the theory of dynamical systems is central to many disciplines and offers a mathematical formalism to characterize complex adaptive systems.

The theory also forms the basis of a field of neuroscience^9^ and facilitates a better understanding of the activity inside the brain. The dynamics inside the central nervous system give rise to complex behavior, enabling the living being hosting the brain to solve challenging problems, and are therefore of great interest to this research field.

Moreover, there is increasing interest in physical systems that can serve as substrates for brain-like computations. There are numerous dynamical systems that perform non-trivial computations on input signals using the interactions between their parts and the dynamics that arise within them^10–13^.

Evaluating the functional capabilities of such input-driven systems is therefore crucial for the study of neural and neuromorphic systems alike. Specifically, it is vital to compare systems quantitatively, to uncover relationships between computations and structural or dynamical features, and to optimize their performance. For this purpose, benchmarking a system’s performance on standard tasks is illustrative but insufficiently informative, as it cannot give a comprehensive profile of the functions conducted.

Therefore, we investigate a system-agnostic metric to quantify the capacity of a dynamical system to perform arbitrary transformations on an input, i.e. its information processing capacity. Originally proposed in^14^, we extend the notion of passive fading memory^15^ to nonlinear transformations of increasing degrees of complexity. In the original formulation, the evaluation of the processing capacity is based on discrete inputs and discrete system states. For continuous time systems we expand discrete inputs to continuous values and compress sections of continuous system states into discrete steps. Based on this, we study the effects the duration of these sections has on the capacity functions computed by the system. Since we need to impose few restrictions on systems when evaluating this metric, it is ideal for analyzing biology-inspired systems, such as spiking neural networks.

We apply the method to systems of gradually increasing complexity and investigate how different parameterisations of the signal encoding influence the outcomes. First, we analyse the time-discrete echo state network (ESN), before we consider the continuous-time Fermi-Pasta-Ulam-Tsingou (FPUT) oscillator chain. With a balanced random network (BRN), we take the step to spiking neuronal networks (SNN), in order to consider a more complex representative of these systems in the next step with a model of a cortical column.

Since there is no generally accepted way to pass real numbers to SNN, we evaluate different methods to achieve this. The signal can be represented by rates of spike trains or by changes in an injected current, it can be spatially encoded or given to all neurons simultaneously, either as a fixed value or multiplied by randomly drawn weights.

Using a direct current signal is motivated by the fact that it represents a simple and precise way of input. However, this mechanism is biologically implausible, since a large part of the communication in the brain takes place via sequences of spikes. To stay close to the biology of the brain and thus increase the probability of revealing insights into its function, it is desirable to rely on signal transmission via spikes. The reasoning behind the encoding methods is similar. The simplest option is to apply the same signal to every neuron. If this input causes too little variability in the network, the next possibility is to weight the inputs to the neurons unequally. However, methods, in which all neurons are stimulated simultaneously, stand in contrast to the biological implementation, in which in most cases only a small part of the neurons is activated. Therefore, from a biological point of view, spatial encoding, which always excites only a small part of the network, is preferable to the other methods.

SNN commonly receive spike trains as background input to bring them to a suitable working point in terms of firing activity and spiking statistics. We need this background activity because we simulate networks that represent a very small part of the brain and cannot be considered as completely independent components. They receive spikes from their environment, which we approximate by random Poisson spike trains. This background noise reduces the processing capacity^14^, as it introduces an additional input that is not taken into account when evaluating the computed transformations of the main inputs. Therefore, we investigate ways to reach an appropriate working point without introducing an untreated input signal.

The information processing capacity^14^ is based on linear encoded inputs. Any nonlinearity in the input encoding distorts the capacity profile and complicates its interpretability. However, there are reasons, such as the desire for biological plausibility, to analyse the system using nonlinear inputs. For these cases, we provide a method to remove the effects of such encodings and provide a lower bound on the actual processing capacity of the dynamical system. This extraction of the encoder capacity allows to investigate parts of complex dynamical systems separately by considering all signals entering the subsystem as encoded by the rest of the system. For example, computations of parts of a comprehensive brain model could be studied separately without isolating them from the overall system.

As a last step we show that the capacity profile correlates with the performances on tasks of different complexity and with different memory requirements and thereby emphasize the usefulness of this metric in the characterization of dynamical systems.

## Results

**Figure 1:**
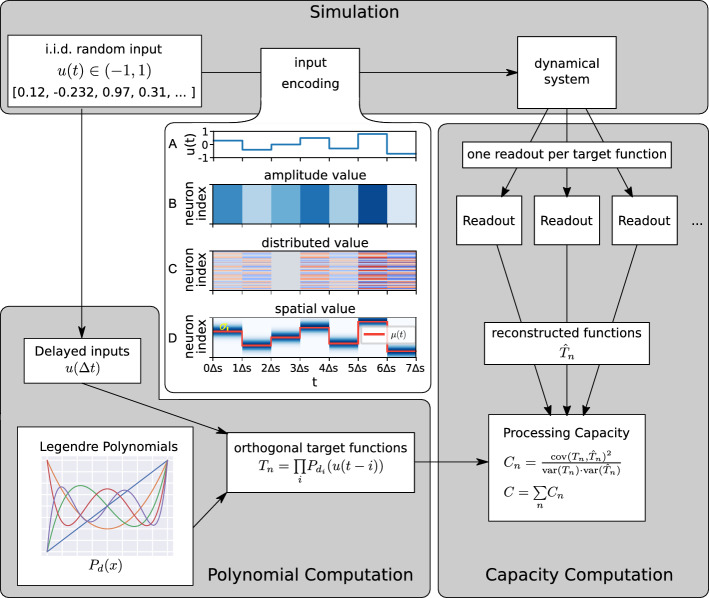
Schematic of processing capacity. *Simulation*: real-valued random inputs between -1 and 1 are encoded and fed into the dynamical system. (**A**) temporally unfolded signal. (**B**) amplitude-value encoding where each neuron receives the same input. (**C**) distributed encoding where the encoder is connected to the system by randomly drawn weights. (**D**) spatially encoded signal. *Polynomial computation*: Products of Legendre polynomials with delayed inputs are calculated as target functions. *Capacity computation*: the reconstructed functions are evaluated against the target functions using the squared correlation coefficient.

We investigate systems of varying complexity (see Section “Investigated models”) to uncover relationships between input and system parameter configurations and their processing capacity. The schematic representation in Fig. 1 shows how the information processing capacity tests the systems’ ability to calculate orthogonal polynomial functions based on different delayed inputs. To provide a comprehensive view of the systems’ computations, we look not only at the total capacities of the dynamical systems, but also at their composition, based on the maximum polynomial degree and maximum input delay (i.e. nonlinearity and memory, see Section “Processing capacity”).

### Discrete time system: echo state network

Our first case study is a discrete-time recurrent neural network, an *echo state network*. This model was chosen to validate the metric implementation because it is neuro-inspired at a fundamental level and due to the simplicity of the system, it allows fast numerical simulations and, consequently, detailed analyses.

**Figure 2:**
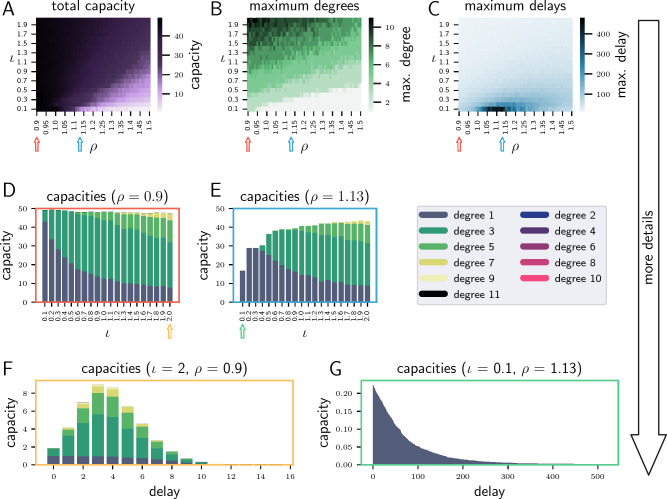
ESN results with increasing level of detail from top to bottom. (**A–C**) Heatmaps of the total capacity, maximum degree and maximum delay for a parameter scan over $\rho$ and $\iota$ and based on all capacity functions (over all degrees and delays). (**D**) and (**E**) Total and degree-specific capacities as a function of $\iota$, for the values of $\rho$ indicated by the correspondingly coloured arrows in (**A–C**). Values are summed over all delays for each bar. Note that the ESN only exhibits capacities of odd degree, because of the odd nature of the tanh nonlinearity. (**F**) and (**G**): Capacity profiles showing total and degree-specific capacities as a function of delay for the parameter configurations indicated by the correspondingly coloured arrows in (**D,E**).

We extend the ESN experiments conducted in^14^ by performing a comprehensive parameter scan, evaluating the maximum degree and delay in addition to the total capacity, looking at more detailed capacity profiles and comparing the capacity properties with task performance. Figure 2 shows the results of the ESN with varying levels of detail and focus on different aspects of the capacity. Panel A shows the total capacity of the ESN as a function of input gain $\iota$ and feedback gain $\rho$ (see Section “Investigated models”), which scale the input and recurrent weights, respectively. For feedback gain values below 1, the total capacity is close to the maximum of 50 for all $\iota$. The capacity decreases for higher values of $\rho$ and low values of $\iota$. However, the processing capacity metric (see Section “Processing capacity”) provides more information about the computations performed by the system. First, we consider the complexity of the computations, expressed by the ability to reconstruct polynomials with higher degrees. Although the changes in the left part of the capacity heat map A ($\rho \le 1$) seem to be minimal, the degree heat map B shows that the ability to perform nonlinear computations increases with $\iota$. This can be accounted for by the shape of the $\rm{tanh}$ nonlinearity. For input values close to zero, it is almost linear, while higher and lower values reach the saturating nonlinearity of the transfer function. Charts D and E illustrate how increasing values of the input gain $\iota$ shift the computational capacity away from linear to increasingly nonlinear computations.

With regards to the delays that are representable by the ESN, i.e. the system’s memory, panel C shows that even in the parameter range with low $\iota$ and $\rho > 1$, which corresponds to low total capacity, there are notable changes in the computations. Compared to the homogeneous values for the maximum delay at higher input gains, the maximum delay increases dramatically with the feedback gain up to $\rho = 1.13$. Thus, these results demonstrate that the system can operate at two extremes - linear, high-memory processing, and nonlinear, low memory processing—as depicted in F and G, i.e. there is a tradeoff between memory and nonlinearity (see Section “Discussion”).

**Figure 3:**
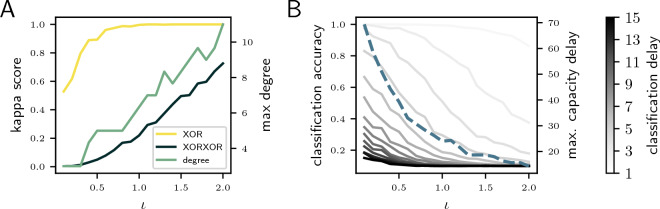
Comparison of ESN task results with the processing capacity as a function of $\iota$. (**A**) Performance for nonlinear tasks (yellow and blue curves; left y-axis) and the maximum capacity degrees (green curve; right y-axis). (**H**) Performance for memory tasks (gray curves; left y-axis) and maximum capacity delay (blue dashed curve; right y-axis).

The method allows us to evaluate the system’s ability to compute nonlinear functions with different memory requirements. To analyze whether these insights hold for structured instead of random input signals, we evaluate the ability of the systems to solve tasks requiring varying degrees of nonlinearity and memory and compare the results with properties of the capacity profiles. Therefore, we compare the maximum capacity degree with the performance in the nonlinear exclusive-or (XOR) task and test the system on a delayed classification task that requires deciding which of ten streams was active before a given number of input steps. We set $\rho$ to 0.9 and examine the performance of the task with varying $\iota$.

Figure 3 visualizes the comparison between the results of the tasks performed by the ESN and the corresponding capacity properties. Panel A shows how the performance of the XOR task (yellow curve) increases along with $\iota$ until it saturates near the maximum value of one. Since the performance is saturated for a large portion of the parameter range, we also evaluate the more complex nested XOR task (XORXOR). The blue curve in A shows that the XORXOR performance of ESNs is also strongly correlated with the maximum degree.

Panel B compares the maximum capacity delay (dashed curve) with the classification performance at different delays (indicated by shades of gray). Increased maximum capacity delay corresponds to an increase in the delayed classification performance. However, the graph shows that the maximum capacity delay is larger than the delay for which the system can still perform the classification. One explanation is that the capacity at the maximum delay is usually small, as shown in Fig. 2F,G. In addition, for the classification not only one but ten input streams with different connection weights are fed into the network. Nevertheless, the results show that the capacity delay is a good indicator for the memory in ESNs.

### Simple continuous time system: Fermi-Pasta-Ulam-Tsingou model

**Figure 4:**
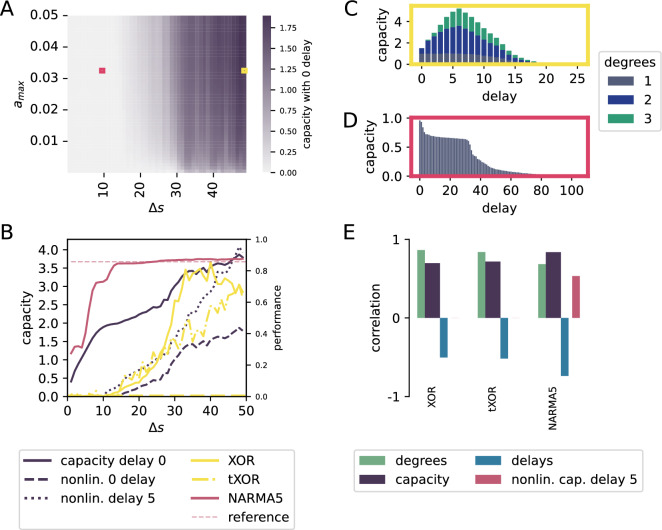
FPUT results. (**A**) Heatmap of nonlinear capacity with zero delay, where the maximum amplitude $a_{max}$ is a force given in arbitrary units, the input duration is given in units of simulation time steps. (**B**) Total nonlinear capacity, nonlinear capacity with zero delay (dashed) and with at least delay 5 (dotted) as well as performance on XOR (yellow), time delayed XOR (yellow, dash-dotted), NARMA5 (maroon) and NARMA5 evaluated on a linear reference system with perfect memory (light maroon, dashed) over input duration for fixed $a_{max}=0.033$. The performance of both XOR tasks is measured using the Cohen’s kappa score and for NARMA it is the squared Pearson’s correlation coefficient. (**C, D**) Capacity profiles showing total and degree-specific capacities as a function of delay for the parameter configurations indicated by the correspondingly coloured squares in **A**. (**E**) Correlations between capacity and task performances for the same parameter ranges of amplitude and duration as shown in (**A**).

The previous section demonstrates that the processing capacity of a simple discrete time system, with respect to complexity and memory, depends on the relative strengths of input and recurrent weights. Here, we extend the analysis to a simple continuous time system, the Fermi-Pasta-Ulam-Tsingou (FPUT) model. We investigate a chain of 64 nonlinearly coupled oscillators; input is applied to all equally. To give discretized input to the chain, we need to decide for what time period we present each input. Panel A of Fig. 4 illustrates the impact of different input amplitudes and durations on the nonlinear capacity with zero delay. Panel B shows the nonlinear instantaneous capacity for fixed $a_{max}=0.033$ as a function of $\Delta s$, compared to total instantaneous capacity and capacity with delay 5. While the total capacity with delay 0 is already non-zero for small step sizes, the nonlinear capacities both remain zero for small $\Delta s$. This is consistent with the system’s improved performance in solving XOR and time-delayed XOR tasks, which exceed chance level ($\kappa =0$) as soon as nonlinear capacities become non-zero.

The NARMA5 performance starts to get close to the performance of a reference implementation of a linear system with perfect memory when the delay 5 capacity becomes non-zero. For large step sizes, the FPUT marginally exceeds the performance of the linear reference system. Interestingly, the nonlinear capacity with delay 5 becomes nonzero for smaller $\Delta s$ than its instantaneous counterpart. This can be understood via panels C and D, which show capacity profiles resolved by delay, revealing that nonlinear capacities grow with time lag before declining again. Even for large $\Delta s$ as in Panel C the quadratic capacity at zero time lag is small compared to the one for delays up to 10, and the cubic capacity remains zero. These higher nonlinear capacities come at the cost of losing ability to reconstruct linear signals with large delays, as seen in the long tail of panel D. Finally, panel E shows the correlation between capacities and task performance. Especially interesting is the negative correlation with delayed capacity for all tasks. This can be again explained by the shape of the capacity profiles: the ability to reconstruct signals with larger delays contribute to neither of the XOR tasks, but yield larger capacities. The NARMA5 task is only sensitive to dynamics with a delay of 5, which is also reflected in the capacity.

### Balanced spiking neural network model

**Figure 5:**
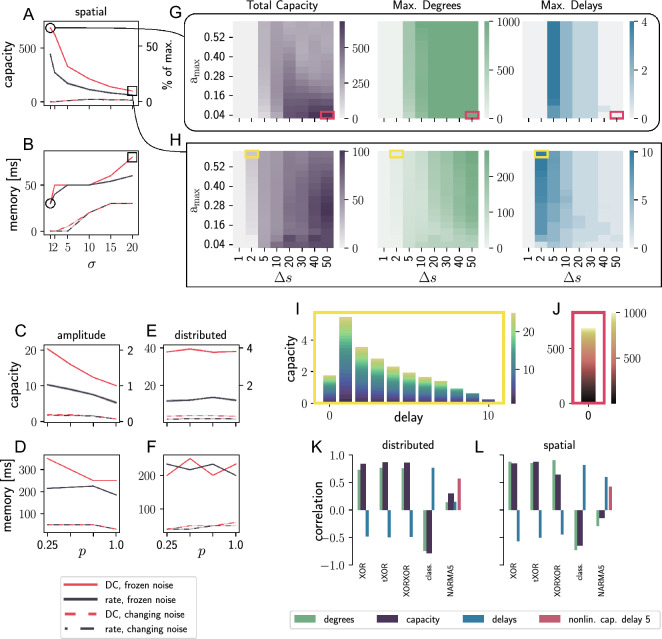
BRN results for different encoding schemes. (**A, B**) maximum capacity and memory (product of maximum delay and step duration) for the spatial encoding scheme averaged over five trials with different network and input initialisations. The standard deviation across trials is too small to be visible. (**C, D**) as for (**A, B**), but with amplitude encoding. (**E,F **) as for (**A, B**), but with distributed encoding. (**G, H**) Heat maps for total capacity, maximum degree and maximum delay for the parameter scans marked with a circle and a square in A and B. (**I, J**) Capacity profiles showing total and degree-specific capacities as functions of delay for the parameter configurations indicated by the magenta and yellow boxes in (**G**) and (**H**) Note the different axis and color scales in these panels. (**K, L**) Correlations between capacity statistic and task performances for the same parameter ranges of $a_{\max}$ and $\Delta _s$ as shown in (**G**) and (**H**), using DC input and frozen noise; $p=1$ in (**K**) and $\sigma = 20$ in (**L**).

Spiking neural networks are continuous time systems that communicate via pulses. Applying the random signal needed for measuring the computational capacity to spiking neurons requires extra steps: we encode the signal with an amplitude-value scheme; a distributed-value scheme or with a spatial-value scheme (see Section “Processing capacity” and Fig. 1). We use a balanced random network (BRN) consisting of two populations of neurons as an entry model for spiking neural networks. We choose this system because it is one of the simplest SNN architectures, while still providing rich dynamics through the interplay of excitation and inhibition (see e.g.^16^).

#### Encoding schemes distribute memory and nonlinearity

Figure 5 visualizes the results of the BRN with different encoding schemes. The different panels focus on distinct properties and details of the processing capacity and its connection to task performance. Panels A-F show the maximum capacity and maximum memory measured for the BRN whilst varying the proportion *p* of input neurons for the amplitude-value and distributed-value scheme and the standard deviation $\sigma$ of the spatial-value scheme, respectively. Each data point in A, C and E corresponds to the maximum capacity resulting from a parameter scan over the maximum amplitude $a_{\rm{max}}$ and the step duration $\Delta s$ of the input signal. B, D and F show the same analysis performed on the maximum memory the network can retain, i.e. the maximum number of delays multiplied by $\Delta s$.

Comparing the positions of the curves in all six plots, we observe that frozen background noise (solid curves) increases maximum capacity and maximum memory over changing noise (dashed curves), see Section “Investigated models”. This is a consequence of the reduced randomness in the system. Likely due to the same cause, the performance is higher for a direct current than a rate input in the frozen noise condition, whereas the input modality plays little role in the changing noise condition. Comparing A, C and E, we conclude that spatial-value encoding results in higher maximum capacities than the amplitude-value and distributed-value schemes. In contrast, the maximum memory (B, D and F) is higher for the amplitude-value and distributed-value encodings.

Within A and C, reducing the number of neurons receiving input in a given input step, i.e. *p* for amplitude-value encoding and $\sigma$ for spatial-value encoding, increases the maximum capacity for the tested parameter range. Conversely, a wider distribution across the input neurons is beneficial for the maximum memory when using spatial-value encoding (B) but plays little role for the amplitude-value encoding (D). However, distributed-value encoding does not show comparable trends. The input connection probability *p* neither significantly changes the total capacity (E) nor the maximum memory (F). The fluctuations in the results are so minor that their standard deviations are too small to be visible in these panels.

In summary, these results suggest that spatial encoding maximizes the system’s ability to compute nonlinear functions whereas amplitude-value encoding maximizes memory, while both perform best with frozen background noise, direct input current and a smaller number of input neurons. Motivated by its superior overall capacity, we show a detailed analysis of the spatial-value approach in G-J. The first row of heat maps shows the total capacity, the maximum degree and the maximum delay as a function of the maximum amplitude $a_{\rm{max}}$ and step duration $\Delta s$, assuming a standard deviation $\sigma =1$ for the distribution of the input over the neurons, which results in the maximum capacity observed in A (circle). The next row shows the equivalent results, but with a standard deviation $\sigma = 20$, which leads to the maximum memory in B (square).

The capacity heat maps for the two input configurations exhibit their maximum capacity at the maximum step duration $\Delta s$ of $50\;\rm{ms}$. However, the best values for the maximum amplitude $a_{\rm{max}}$ are different for each $\sigma$. While the system with $\sigma = 1$ has its maximum at a low input amplitude of $0.04\;\rm{pA}$, we need to increase $a_{\rm{max}}$ to $0.24\;\rm{pA}$ for $\sigma = 20$ to reach the maximum capacity. The green heat maps show that longer step durations cause higher maximum degrees. The heat maps displaying the maximum delays show similar trends, i.e. a decrease in the maximum delay as we increase the step duration, which we attribute to the increase of absolute time the network has to maintain the information of a previous step with prolonged step durations.

I and J illustrate capacity profiles for a high capacity parameter set and a high delay parameter set. Together with their positions in G and H (yellow and magenta markers), they show that these networks obtain their total capacity value based on very different compositions of target functions. The yellow framed profile consist of many functions based on delayed inputs, while in J the computations are invariably based on the undelayed signal and consist instead of very high degree nonlinear functions.

#### Correlation with task performance

To assess whether we can infer information about task performance from processing capacity, we calculate the correlations between task scores and total capacity, maximum delay and maximum degree, respectively. The bar graphs at the bottom of Fig. 5 show the results for distributed encoding with $p=1.0$ (K) and spatial-valued encoding with $\sigma =20.0$ (L). XOR, tXOR and XORXOR (see Section “Tasks”) show high correlations with maximum degree and total capacity for both encoding types, while the correlation with the performance of the XORXOR task and total capacity is lowest for the spatial encoding setup. The maximum delay is positively correlated with the maximum classification delay in both cases. The opposite correlation values for the maximum degree and maximum delay (also observed in the other systems) indicate a trade-off between nonlinear computation and memory.

The NARMA5 task^17^ requires both memory and nonlinear computation. The bars for this task in K and L show lower correlations with capacity, delay and degree compared to other tasks. To account for the dependence on the combination of memory and nonlinear computation, we also evaluate the correlation of NARMA5 task performance with nonlinear capacity at delay 5, resulting in a higher correlation for the distributed encoding setup.

#### Encoding effects

**Figure 6:**
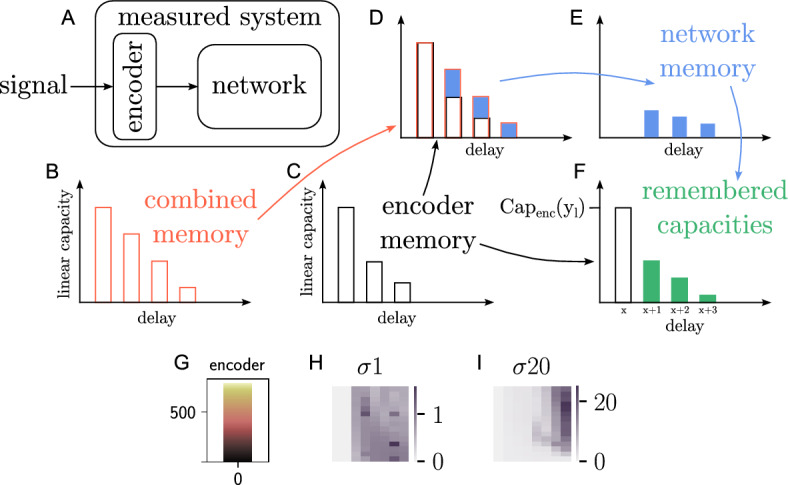
Schematic for the calculation of remembered encoder capacities. (**A**) Diagram emphasizing that the combination of encoder and network acts as the measured system. (**B**) Linear memory of the combined measured system. (**C**) Linear memory of the encoder. (**D, E**) Difference between (**B**) and (**C**) that results in the actual linear memory of the network. (**F**) Possibly nonlinear encoder capacity of target function $y_l$ (black border) and its versions remembered by the network (green). (**G**) capacity profile for the encoder used in Fig. 5J. (**H, I**) Capacity heat maps as in Fig. 5G,H, but with all possible encoder capacities subtracted. Note the different scales of the color bars.

Despite being an important component of signal processing in (bio)physically meaningful systems, the encoding scheme can bias the capacity estimation. As Panel A of Fig. 6 illustrates, the effectively measured system includes the effects of the encoder, although we are only interested in isolating the system itself. The main system can have nonlinear capacities without calculating nonlinear functions itself by merely remembering the nonlinear or delayed inputs from the encoder, i.e. the encoder can introduce nonlinearity and memory which bias the estimate. One solution are linear encoders without memory. However, when we prefer more complex methods to encode the signal, for example to increase biological plausibility, we want to exclude encoder effects from the measured capacity. Figure 6B–F outline a way to calculate the encoder effects that need to be subtracted.

We first calculate the capacity of the encoder output by building the state matrix from the encoded signals that the neurons get as inputs, instead of taking the membrane potentials to evaluate the processing capacity. In the case of the spatial value scheme, this encoder state matrix is built from the Gaussian profiles representing the inputs (see Fig. 1D for a time-unfolded version of such a state matrix). With these capacities and the capacities of the overall system we calculate the main system’s effective linear memory by subtracting the encoder memory (degree 1 encoder capacities) from the combined memory (based on the membrane potentials) for each delay (B-E). Based on the system and encoder memory values, we calculate the fraction of the encoder input the system can memorize after each delay. Using these fractions, we compute how much of a capacity value for a target function can be based on remembering a target function that is already computed by the encoder. These remembered capacities (F) result from the linear memory of the system and the nonlinearity and memory of the encoder. Therefore we subtract them from the capacities of the combined system to obtain the effective capacity of the main system for all objective functions (see supplementary materials).

With this method we cannot get information about the precise functions the system computes as with linearly encoded input, because the system computes the function $F_{\rm{sys}}(F_{\rm{enc}}(u))$ instead of $F_{\rm{sys}}(u)$. Therefore a system capacity $C^{\rm{sys}}(y_l) > 0$ does not mean that the system computes the specific target function $y_l$ with the degree $d_{y_l}$. However, it tells us that the system computes a function which goes beyond remembering the input.

A problem with this procedure is that the capacity values cannot necessarily be subtracted, divided and multiplied, but need to be transformed first to get the correct system capacity. These transformations can be different for every target function and we need additional information to calculate them correctly (see supplementary materials). However, we can subtract all encoder capacities and their delayed versions completely, i.e. we fully remove all capacities of target functions that can be partly a result of the network remembering the encoder inputs. This results in a lower limit for the total capacity that can be calculated for any type of encoder and dynamical system. However, it depends on the scientific question whether it makes sense to remove the encoder capacities. For example, if the aim is to compare the information processing capacity with the performance on tasks whose inputs are encoded in the same way, it makes more sense to analyze the capacity of the entire system. If the intention is to examine the calculations of the dynamic system independently of the encoder calculations, then the encoder capacities should be subtracted. For more details on the procedure and the different ways to remove the encoder capacities, see the corresponding section in the supplementary materials.

Panel G shows that for the spatially encoded signals the encoder alone has higher capacities than the network in Fig. 5J. H and I show the lower limits for the capacity functions actually calculated by the network. Removing the encoder capacities leaves only a fraction of the original results for $\sigma = 1$. In contrast, networks given a wider input with $\sigma = 20$ compute target functions beyond those remembered from the input. Apart from that, the delays remain unchanged because the spatial value encoding does not introduce any additional memory into the system.

### Biophysical spiking network model

**Figure 7:**
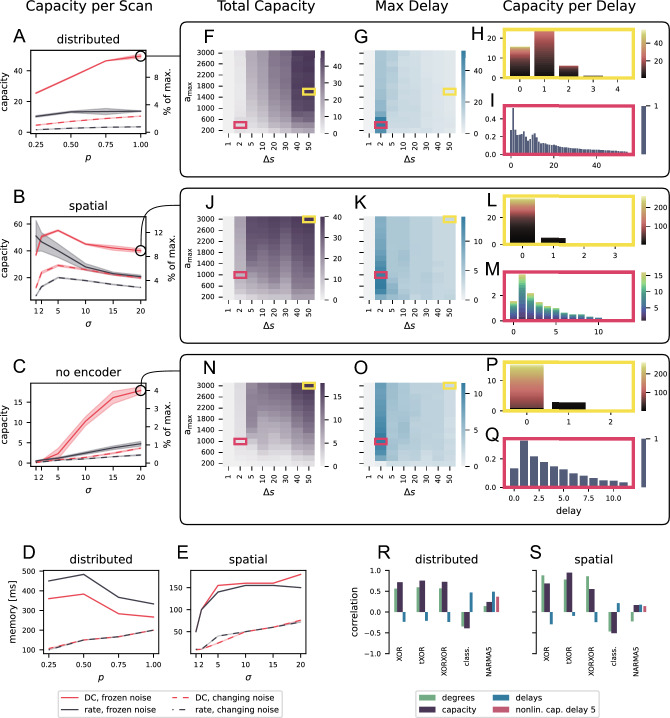
Microcircuit results for different encoding schemes. (**A–C**) Maximum capacity per input parameter *p* or $\sigma$ respectively for distributed encoding (**A**), spatial encoding (**B**) and spatial encoding without remembered encoder capacities (**C**) averaged over five trials with different network and input initialisations. Shaded areas indicate the standard deviation across trials. (**D, E**) Maximum memory (product of maximum delay and step duration) for distributed and spatial encoding. (**F–Q**) Capacity (**F, J, N**) and delay (**G, K, O**) heat maps of the parameter scans corresponding to the markers in (**A–C**) together with capacity profiles for a high capacity (yellow) and high delay (magenta) parameter configuration. Note the different axis and color scales in the bar graphs. (**R–S**) Correlations between capacity statistic and task performances for the same parameter ranges of $a_{\rm{max}}$ and $\Delta _s$ used in (**F**) and (**J**), both with DC input and frozen noise; $p=1$ in (**R**) and $\sigma = 20$ in (**S**). .

We analyze the processing capacity of a biophysically more detailed spiking microcircuit model^18, 19^ by using two encoding schemes: distributed encoding and spatial-value encoding. We chose this network because it combines neurobiological properties such as a data-based connectivity and synaptic plasticity in a network size that still allows for extensive simulations and analyses. Figure 7 compares the different encoding schemes for the microcircuit model at different levels of processing capacity detail. Panels A-E show the summarized results for the different encoding schemes. There are strong similarities to the BRN, in particular: 1) the use of frozen instead of changing noise increases computational capacity and memory; 2) spatial coding is superior to distributed coding when we do not subtract the encoder effects, but gives lower capacities otherwise; 3) DC input leads to higher capacities in most cases than rate encoded inputs. There is one exception to this last point, namely the spatial-value encoded signals with very short stimulus duration, and this effect disappears when we remove all remembered encoder capacities (C). Although there are some larger standard deviations (shaded areas) than in the BRN, the overall variation in capacity results is still small.

Although the microcircuit is less uniformly structured and includes synaptic short-term dynamics, the maximum total capacity is lower than for the BRN, not only in absolute values (BRN: 686, MC: 49.5), which could be explained by the smaller network size, but also when we consider the normalized capacity values (right axis of A–C; BRN: 68.6 %, MC: 11 %). These values are the fraction of the number of readout neurons, since this is the upper limit for the capacity. Each of these maximum values are based on the spatial encoding scheme. However, if we compare only the normalized capacities for the distributed encoding, they are around 4 % for both networks. Moreover, if we subtract the remembered encoder capacities from the spatial encoding values, the microcircuit (4%, Fig. 7C) outperforms the BRN (2.5%, Fig. 5L). In addition, the microcircuit can store information about past inputs longer than the BRN (D and E; BRN: 350 ms, MC: 483 ms). This is likely due to the synaptic plasticity and the biologically inspired connectivity structure, as these features were shown to lead to longer memory in^18^ and^19^.

Figure 7F–Q give details of the parameter scans for distributed encoding, spatial-value encoding and spatial-value encoding with removed encoder effects, respectively. The heat maps for total capacity and maximum delay show that longer input durations tend to correspond to higher capacities based only on short delays and thus higher degrees, while shorter steps allow longer delays. We show detailed capacity profiles with high degrees but small maximum delays (yellow frames) and lower maximum degrees but longer delays (magenta frames) in the bar graphs on the right of Fig. 7. The differences between the two calculation modes are clearly visible, but in contrast to the results for the BRN, here even the configurations with high degrees exhibit memory.

As for the BRN, we evaluate the correlation between the different capacity statistics and the performance on tasks. Figure 7R–S shows the two configurations marked in A and B. As expected, capacity and degree are positively correlated and the delay is negatively correlated with the performances of the nonlinear tasks (XOR, tXOR, XORXOR). The opposite is true for the memory based task, i.e. delayed classification (class.). Overall, however, the correlations with the spatially encoded tasks (S) are lower for the microcircuit than for the BRN, especially for the memory tasks, and even the nonlinear capacity for the NARMA5 specific delay 5 (dark pink) does not improve the correlations.

### Comparative performance on tasks

**Table 1:** Performance on tasks and capacity measures for different dynamical systems. Given as Cohen’s kappa score for XOR, tXOR and XORXOR, maximum delay up to which accuracy is above chance level for delayed classification, and squared correlation coefficient for NARMA5.

	ESN $\rho$ 0.9	FPUT $\alpha$ 0.25	BRN distr. p 1	BRN spatial $\sigma$ 20	MC distr. p 1	MC spatial $\sigma$ 20
Readout units	50	64	1000	1000	447	447
XOR	1.	0.85	1.	0.99	1.	0.99
XORXOR	0.73		0.17	0.62	0.21	0.22
tXOR		0.72	1.	0.99	1.	0.99
Classification			26	18	42	50
NARMA5		0.88	0.29	0.2	0.43	0.17
Capacity	49	55	38	100	49	40
Max. degree	11	3	35	285	71	255
Max. delay	69	688	32	10	53	14

Table 1 shows the maximum task performances of each system, and its corresponding capacity measures. XOR and tXOR are almost perfectly solvable for the investigated systems except for the FPUT, whereas XORXOR is difficult for all systems. ESN and the BRN with spatial encoding stand out with significantly higher XORXOR values than the other systems. The microcircuit benefits from its connectivity and short-term plasticity in delayed classification, as its measured delay is significantly higher compared to the BRN. The results of the NARMA5 task in combination with the maximum delays and the maximum degrees show that long memory is more important than nonlinear computations for solving this task. Therefore, especially the FPUT oscillator chain and the microcircuit with distributed encoding perform better than the other models.

## Discussion

The information processing capacity enables thorough investigations of dynamical systems in terms of the functions they can compute. It produces a comprehensive computational profile with intuitively interpretable indicators of complexity (polynomial degree) and required memory (maximum delay).

We explored ways of applying the information processing capacity to dynamical systems with increasing complexity, culminating in and focusing on biologically inspired spiking neural networks. Our initial experiments extend the analysis of the (discrete-time) ESN used in^14^. By investigating the FPUT model, we expand the scope of our study to continuous-time systems, and then make the step into SNNs with a balanced random network. Finally, we apply the measure to a cortical column model.

We evaluate the effects of different input parameterisations to provide a guide for future application of the capacity measure to similar systems and especially to spiking neural networks. Although the metric is highly informative, it can be computationally expensive and any restriction on the parameter search space drastically saves computational costs.

As reported in previous work such as^20^ and^14^, we found no single optimal parameterisation that simultaneously maximizes nonlinearity and memory capacity. All dynamical systems show a trade-off between memory and nonlinear processing, since their processing capacity is bounded by the number of readout units. Therefore, for a given use case, one would have to tune the parameters appropriately to achieve optimal results. For continuous-time systems, an increase in step duration is accompanied by a shift to the nonlinear regime. Our explanation is that the systems have more time to transform a single input step, but in return they also need to retain information for a longer period to process previous signals. The ESN has a small parameter range that results in particularly long memory. In this range, the input scaling is so small that the signal is transformed mainly by the linear part of the tanh activation function, while a spectral radius slightly larger than one ensures a strong influence of previous inputs.

A reduction of capacity by introducing noise into the dynamical system has previously been reported by^14^. Consistent with these findings, we also find that sources of randomness commonly used in spiking neural networks, such as driving the network with Poisson spike trains and encoding the inputs as firing rates of such spike trains, reduce the processing capacity. We therefore propose to first operate the systems deterministically using frozen background noise and direct current as inputs, and later to analyse the robustness to random perturbations as a separate property.

While the ESN for most parameters has near-maximum capacity, there are both high and low capacity configurations for the FPUT. In contrast, the SNNs achieve only a fraction of the possible capacity. We note that in our study, the information processing capacity or different aspects of it, such as the maximum degree and delay, generally show strong correlations with task performance. Whereas even systems whose processing capacity is low can solve tasks such as XOR or tXOR almost perfectly, higher capacity systems such as the ESN have a clear advantage for more demanding tasks such as XORXOR. However, previous research has shown that the capacity may not be a reliable indicator of a system’s ability to predict the states of dynamical systems that evolve independently of external inputs^21^.

An explanation for the low capacity of SNNs may be the frequent reset of the membrane potential after a spike, especially since we use the membrane potential as a state variable for the readout. If these results are indicative of the computational power of biological neural networks, and under the assumption that the information processing capacity captures a large portion of the relevant information processing that dynamical systems, including the brain, can perform, this is an indication against the reservoir computing approach as a tool for determining (and a model for understanding) the computational power of cortical systems. However, higher performance can probably be achieved by using more complex neuron models and heterogeneous parameter distributions, as these adjustments have positive effects on computational performance^22^. As we do not focus on finding a network with maximum capacity, but on adapting the capacity metric for the general application to SNNs, we leave this investigation for future work.

We must encode the signal linearly to prevent a distortion of the capacity results. With nonlinear encoding, systems can reconstruct polynomial functions by remembering the nonlinear inputs over several time steps without transforming them. Thus, we advise to use only linear encoders if possible.

However, there are reasons to use nonlinear inputs such as the desire for biological realism in models in computational neuroscience. Therefore, we presented a procedure to remove nonlinear encoder effects and provide a lower bound on the main system’s actual capacity. The procedure can also handle encoders with memory. This enables, for example, to analyse specific parts of larger systems, for example models of the brain, separately. For this purpose, we consider all components feeding signals into the subsystem as part of the encoder and remove their capacity from the results.

In its current form, this method only provides a lower bound of the capacity. Further research could develop a more precise removal of the encoder effects that includes additional metrics gathered during the reconstruction of the individual target functions. In addition, due to the high computational costs, which increase quadratically with the system size, an investigation of possible effects of subsampling methods or the dimensional reduction of the state matrix on the expressiveness of the capacity can offer additional value.

Overall, we have laid the foundation for detailed analyses of various systems , encompassing discrete and continuous-time systems as well as biologically inspired neural networks. This method can now be used, for example, to test computational hypotheses on spiking neural networks and even in-vitro experiments, or to optimize the parameter configuration and input encoding of neuromorphic hardware for a given computational goal.

## Methods

### Processing capacity

Initially proposed by^14^, the empirical estimation of information processing capacity of a dynamical system quantifies the different processing modes it can employ by determining the number of linearly independent functions of its input that the system can compute. Figure 1 shows a schematic representation of the metric, see also^14^. The dynamical system under investigation is passively driven by a time-dependent input signal *u*(*k*) of finite total length *T*. In this work the discrete values of *u*(*k*) are independent and identically drawn (i.i.d.) from a uniform distribution over the interval [$-$1, +1]. The aim of the analysis is to quantify the system’s ability to carry out computations on *u*. For that purpose, we gather the system’s states in response to the input *x*[*u*(*k*)], train linear estimators using the Moore-Penrose pseudoinverse to reconstruct a set of *L* target functions $y_l$ for $l = 1,..., L$ from these states and evaluate how well the system is able to reconstruct each function by calculating the squared correlation coefficient between the target functions $y_l$ and the reconstructed functions $z_l$:

1 $$\begin{aligned} C_l = \frac{\rm{cov}(y_l, z_l)^2}{\rm{var}(y_l) \cdot \rm{var}(z_l)} \end{aligned}$$

The sum of the single capacities $C_l$ over all target functions is the total processing capacity of the system.

We choose the target functions to be orthogonal to the input distribution. This orthogonality of the target functions ensures that each independent measurement reflects an independent transformation that the system can perform. Furthermore, we compute target functions not only based on *u*(*k*), but also on time-delayed versions $u(k-i), \forall i \in [0, k]$, in order to measure the amount of linear and nonlinear memory the system can maintain. The basic components of the orthogonal target functions that we use in this work are Legendre polynomials, which are defined by

2 $$\begin{aligned} {\mathcal {P}}_{d}(s) = \frac{1}{2^{d}} \sum _{j=0}^{d} \bigl ({\begin{matrix} d \\ j \end{matrix}}\bigr )^{2} (s - 1)^{d-j} (s + 1)^{j} \end{aligned}$$

where *d* is the degree of the polynomial. Each unique target function is composed by selecting a degree $d_i$ for each considered delay *i* and building the product of the thereby specified polynomials:

3 $$\begin{aligned} y_l = \prod _{i} {\mathcal {P}}_{d_{i}} (u[k-i])) \end{aligned}$$

where ${\mathcal {P}}_{d_i}$ are the Legendre polynomials of degree $d_i$. Thus the degree tuple $D_l=(d_{l,0}, d_{l,1}, d_{l,2},\ldots , d_{l,m})$ completely defines a target function $y_l$ having a maximum delay $i=m$. The sum of all degrees in $D_l$ determines the total degree of the corresponding target function $y_l$. For example, the degree tuple (2, 1, 0, 4) corresponds to the target function:

4 $$\begin{aligned} y = {\mathcal {P}}_2(u[k]) \cdot {\mathcal {P}}_1(u[k-1]) \cdot {\mathcal {P}}_4(u[k-3]) \end{aligned}$$

which has a total degree of 7. Note that we do not explicitly include the polynomial for delay $i=2$, because its degree is 0 and therefore corresponds to the constant polynomial ${\mathcal {P}}_0 = 1$.

The total degree of a target function specifies the complexity of the required computation (nonlinearity), whereas the delays *i* specify the memory requirements of the investigated system for further details, see^14, 22^.

Since the number of functions in the capacity space is infinite and the computational resources are finite, we cannot evaluate the capacities for all basis functions. Moreover, we do not know in advance which functions the dynamical systems can compute, and therefore we use an exploration strategy to guide our search for nonzero capacities in the space of target functions, rather than determining in advance which ones we will evaluate. The basis of this strategy is the assumption that capacities decrease with the complexity of the target functions and the memory required to compute them. We increase the degree and delay separately and stop computing more functions of higher degree or longer delays when the capacity falls below a threshold whose value is on the order of $O(\frac{N}{{T}})$, where *N* is the number of readout states per input value. For more information on the calculation of this threshold, see the supplementary material of^14^.

#### Input encoding for spiking networks

Different dynamical systems may impose very different (physical) constraints on how a stimulus is encoded. To pass the signal *u*(*k*) to continuous time systems, we thus need to encode this input appropriately depending on the system’s specifications. To do so, we first define which sub-set of units are to receive input, i.e. we specify the density of input connections as a fixed connection probability *p* (see below for model-specific definitions of the set of potential input units). This results in a random subset of $N_{\rm{inp}}=p N$ units that are effectively input-driven. We then define a continuous version of the discrete signal *u*(*k*) in a similar way as in^23^ and^24^, i.e.

5 $$\begin{aligned} u(t) = u(k)\, \rm{for}\, (k-1) \cdot \Delta s \le t < k \cdot \Delta s \end{aligned}$$

where $\Delta s$ is the stimulus step size and $0 \le t < T\Delta s$, see Fig. 1A.

This continuous, time-varying signal is delivered to the system following three different types of encoding schemes. These were chosen due to the constraints of the systems under investigation, but are general enough to cover a wide variety of possible encoding mechanisms in input-driven, continuous, dynamical systems. For the *amplitude-value* scheme (Fig. 1B), the value of *u*(*t*) is encoded by amplitude, i.e. *u*(*t*) to values in the range $[0, a_{\rm{max}}]$ resulting in the scaled amplitude signal:

6 $$\begin{aligned} a(t) = \frac{(u(t)+1)}{2} \cdot a_{\rm{max}} \end{aligned}$$

This amplitude signal *a*(*t*) is also the basis for the *distributed-value* scheme illustrated in Fig. 1C. Here, encoding variability is introduced by drawing the input weights from a uniform distribution in $[-1, 1]$, i.e. *a*(*t*) gets multiplied by a randomly drawn, target-specific weight $w_j$ such that each target neuron *j* receives a slightly different input.

7 $$\begin{aligned} a_j(t) = a(t) \cdot w_j \end{aligned}$$

The *spatial-value* scheme (Fig. 1D) assumes the input can be spatially encoded, i.e. the target neurons have amplitude-specific receptive fields. The scheme thus considers different subsets of target neurons are responsive to stimuli of different amplitudes. The value of *u*(*t*) is considered the centre of a Gaussian profile which determines which units in $[1,\ldots ,N_{\rm{inp}}]$ receive the input. This encoding scheme is illustrated in the bottom panel of Fig. 1. Under this encoding scheme, each input neuron *j* receives an input of amplitude:

8 $$\begin{aligned} a_j(t) = a_{\rm{max}}\frac{\rm{exp}(-j^2/2)}{\sigma \sqrt{2 \pi }}\left( \frac{j-\mu (t)}{\sigma }\right) \end{aligned}$$

where $\mu (t)$ is the distribution’s mean

9 $$\begin{aligned} \mu (t) = \frac{(u(t)+1)}{2} \cdot N_{\rm{inp}} \end{aligned}$$

and the standard deviation $\sigma$ is an experimental parameter, which determines the spatial spread (and overlap) of the input encoding. Note that we do not use periodic boundaries, in order to be able to distinguish the encoding of $u(k)=1$ and $u(k)=-1$.

Note that for this encoding scheme to target a sufficiently large subset of neurons, we clip the input connection density to $p=1$.

The continuous activity signals $a_{\rm{j}}(t)$ are converted into input for spiking neural networks in two ways^25^. In the first method, we interpret $a_{\rm{max}}$ as a maximum current, and supply each neuron with a corresponding scaled direct current $a_j(t)$. In the second method, $a_{\rm{max}}$ is interpreted as the maximum firing rate of a Poisson generator. Thus, *a*(*t*) is converted into the piece-wise rates of an inhomogeneous Poisson generator providing independent spiking input to the input neurons.

### Tasks

We let the dynamical systems solve various tasks and encode the required inputs as close as possible to the way we described for the capacity in the previous section. Continuous inputs (see NARMA task in Section “Tasks”) are encoded exactly like the inputs for the capacity calculations. For multidimensional binary inputs, we need to adjust the encoding. In distributed-value encoding, *n*-dimensional inputs are split into *n* input streams that are separately connected to the system (each with its own connectivity matrix). The value of the activated input streams is set to $a_{\rm{max}}$ and the value of the inactivated inputs is set to 0. In spatial-encoding, each active input is encoded by a Gaussian profile (see Eq. (8)) with a fixed value for its mean. The mean values of the *n* inputs are evenly distributed over the input space and inactive streams do not cause any activation of the system.

#### XOR task variants

As representatives of tasks requiring nonlinear computations, we use exclusive-OR (XOR) and variations thereof. XOR is based on two binary inputs and results in 1 if the inputs are different and in 0 if they are not. A more complex version is the nested calculation of XOR tasks (XORXOR). Unlike the normal XOR task, we need four input signals to perform this task:

10 $$\begin{aligned} \mathrm {XORXOR(inp_1, inp_2, inp_3, inp_4)} = \mathrm {XOR(XOR(inp_1, inp_2), XOR(inp_3, inp_4))} \end{aligned}$$

The temporal XOR task (tXOR) is based on a single input stream and requires additional memory. We define tXOR as the XOR of the current input and the input of the previous time step. We evalutate all XOR variants with Cohen’s kappa score.

#### Delayed classification

In this task, we use ten different input signals, only one of which is active in each step. We then evaluate for how many delay steps in the past a system can identify above chance level which of these ten one-hot encoded signals was active.

#### NARMA time series

The nonlinear autoregressive moving average (NARMA) task tests systems for both nonlinear computation and memory, and its evolution is described by the following equation:

11 $$\begin{aligned} y(t+1) = \alpha y(t) + \beta y(t-1) \left( \sum ^{n-1}_{i=0} y(t-i) \right) + \gamma u(t-n+1) u(t) + \epsilon \end{aligned}$$

We use $(\alpha , \beta , \gamma , \epsilon ) = (0.2, 0.004, 1.5, 0.001)$ and time lag $n=5$ as parameter values and evaluate the results with the squared correlation coefficient.

### Investigated models

#### Echo state network model

The echo state network (ESN), which^14^ also used as an example, is a simple, discrete-time recurrent neural network. The evolution of its $N=50$ state variables $x_i$ is given by:

12 $$\begin{aligned} x_i(k+1) = \rm{tanh}(\rho \sum _{j=1}^N w_{ij}x_j(k) + \iota \nu _i u(k)) \end{aligned}$$

where $\rho$ is the feedback gain that scales the recurrent weights $w_{ij}$ from unit *j* to unit *i*, and $\iota$ is the input gain that scales the weights $\nu _i$ from input *u*(*k*) to unit *i*. We initialize both the recurrent weight matrix and the input weights with values drawn from a uniform distribution between $-1$ and 1. In addition, we orthogonalize *w* and scale it to unit spectral radius. To calculate the processing capacity, and given that this system is used as a baseline, we consider all N units receive the input directly, without further scaling or input encoding, as shown in Eq. (12). For the capacity experiments in this work we use $T=100,000$ input steps.

#### Fermi-Pasta-Ulam-Tsingou model (FPUT)

The Fermi-Pasta-Ulam-Tsingou model describes a one-dimensional string of coupled oscillators and was originally studied to investigate the equipartition of energy among its degrees of freedom^26^. As linear forces between neighboring oscillators lead to an analytically solvable system, several nonlinear interactions were studied in the original paper. For our purposes, it is sufficient to consider quadratic nonlinearities, as they appear in the $\alpha$-FPUT model.

In contrast to the original paper, however, we are not interested in a closed system, but an input-forced system. We drive the system via an external time-dependant field *a*(*t*) coupling to all oscillators equally. Different inputs are then represented via different values of *a*, whereby we ensure $\langle a(t)\rangle _t =0$, i.e. we consider an amplitude-value encoding scheme and a dense input connectivity, $p=1$. To deal with the resulting energy put into the system we also introduce a dampening term with decay constant $\tau$.

These considerations lead to the following differential equation for the oscillators

13 $$\begin{aligned} \ddot{x}_i = (x_{i+1} + x_{i-1} - 2 x_i) + \alpha ((x_{i+1}-x_i)^2 - (x_{i-1}-x_i)^2) - \frac{\dot{x}_i}{\tau } - a(t), \end{aligned}$$

where $x_i$ is the deflection of oscillator *i* from the rest position. For the experiments we used $\alpha =0.25$, $\tau =10$ and $T=100,000$ input steps.

#### Balanced random network model

The simpler spiking neural network we use in this study is a balanced random network (BRN)^16^ consisting of $N = 1250$ leaky integrate-and-fire neurons, sub-divided into a population *E* of $N_{\rm{exc}} = 1000$ excitatory neurons and a population *I* of $N_{\rm{inh}} = 250$ inhibitory neurons. The connections between these neurons are crucial for the functionality and dynamics of the network. Excitatory neurons propagate activation to other neurons, while inhibitory neurons function to regulate this activation, preventing potential runaway excitation within the network. The interplay between these two types of neurons contributes to the balance that allows the network to perform complex computations while maintaining stability. After the initialisation of the membrane potential $V_{\rm{m}}$ with values drawn from a uniform distribution between $V_{\rm{min}}$ and $V_{\rm{max}}$, the neuron dynamics follows

14 $$\begin{aligned} \tau _{\rm{m}} \frac{dV_{\rm{m}}}{dt} = - (V_{\rm{m}} - E_{\rm{L}}) + \frac{\tau _{\rm{m}}}{C_{\rm{m}}}I(t) \end{aligned}$$

where $\tau _{\rm{m}}$ is the membrane time constant, $C_{\rm{m}}$ is the membrane capacitance, $E_{\rm{L}}$ is the resting membrane potential and *I*(*t*) is the synaptic current. Synaptic transmission in this model is considered to elicit a delta-shaped post-synaptic current:

15 $$\begin{aligned} I(t) = \Sigma {w_{ij}\delta (t-t^{\rm{sp}}_{j}+d)} \end{aligned}$$

where $t^{\rm{sp}}_j$ is the time at which neuron *j* spikes and *d* is the synaptic delay. When the membrane potential reaches a fixed threshold $V_{\rm{th}}$, it is set back to the reset potential $V_{\rm{reset}}$ and stays at this value for a refractory period $\tau _{\rm{ref}}$. Recurrent connections among excitatory and inhibitory populations are established to maintain fixed in-degrees of $C_{\rm{exc}}$ (excitatory synapses) and $C_{\rm{inh}}$ (inhibitory synapses). To compensate for the effect of the larger excitatory population, the weight $w_{\rm{inh}}$ of inhibitory synapses is *g* times stronger than the excitatory one $w_{\rm{exc}}$.

In order to obtain responsive networks that operate in biologically meaningful regimes (see^16^), an additional background input is necessary. Besides the input signal *u*(*t*) described in Section “Processing capacity” that we potentially give to all excitatory neurons (based on connection probability *p*), every neuron in this model is driven by time-varying spikes that are connected with the same weight $w_{\rm{exc}}$ as excitatory recurrent synapses and exhibit a firing rate $\nu _{\rm{noise}}$. We use two different methods to generate these background spike trains. In the changing noise version, we use random Poisson spike trains whose spike times vary for each input step. Since we must consider each source of randomness that varies for each step as an additional input to the system that reduces the maximum possible processing capacity, in the frozen noise experiments we generate a single Poisson spike pattern of length $\Delta s$ per neuron and repeat it for each step. As readout values for the capacity calculation, we use the membrane potentials of all 1000 excitatory neurons to represent the state of the system after each of the $T=200,000$ inputs. Table S1 in the supplementary materials lists all parameter values for this network.

#### Cortical microcircuit model

The more complex spiking neural network used in our analysis is the cortical microcircuit model introduced by^18^ and subjected to additional investigation by^19^. This is a network of 560 spiking neurons divided into three layers representing the cortical laminae L2/3, L4 and L5. Each layer consists of an excitatory and an inhibitory population, which are linked across all layers by synapses with short-term dynamics. In the original study, the network consists of conductance-based Hodgkin-Huxley neurons equipped with an additional intrinsic noise mechanism. However^19^, show that even significantly simplified integrate-and-fire neurons do not harm the computational performance of the network and can even increase it. We take advantage of this finding and use this simpler neuron model in the current study, which gives us the benefit of a significantly reduced simulation time. For comprehensive implementation details of the model and a complete parameter specification, see^19^.

We use all neurons that are not part of the inhibitory populations of layers 2/3 and 5 as input neurons and feed the signal described in Section “Processing capacity” into these units. The reason for this choice is that in the original model description all but the two mentioned populations are connected to external inputs. Just as with the balanced random network described above, we also drive this system using spike generators that generate either changing Poisson spike trains or frozen noise. In this case, we use two input streams consisting of 40 spike trains connected to the network in the same way, i.e. using the same synaptic weights and connection probabilities, as the two input streams in^19^. Since we have an additional signal driving the activity as described above, we halved the firing rate of the spike trains to 10 spikes per second compared to the original inputs. To calculate the capacity, we use the membrane potentials of all 447 excitatory neurons at the end of each of the $T=100,000$ inputs.

### Capacity chance level and cut-off value

The theory of the information processing capacity is based on inputs of infinite length. Since we have to use finite inputs in our experiments, we must account for a systematic error in the measured capacities. The chance level of the capacity is given by a chi-squared distribution $\chi ^2(N)$ with mean $\frac{mN}{T}$ and variance $\frac{2vN}{T^2}$ (for details see^14^). *m* and *v* are equal to 1 for independent state variables. However, since we cannot assume the independence of the state variables, we do not know the correct values for *m* and *v*. To define a suitable threshold *c* below which we set all capacities to 0, we calculate the value $c_{\rm{ind}}$ for which the probability $P(\chi ^2(N) \le c_{\rm{ind}}) = 10^{-4}$ for $m=1$ and $v=1$. We account for the unknown factors *m* and *n* by multiplying $c_{\rm{ind}}$ by a constant factor. For the experiments in this article, we use the factor 6:

16 $$\begin{aligned} c = 6 \cdot c_{\rm{ind}} \end{aligned}$$

## Supplementary Information

Supplementary Information.

## Data Availability

The source code and datasets generated during the current study are available in a Zenodo repository^27^, https://www.zenodo.org/record/7688574.
