# Supplementary material for: A refined information processing capacity metric allows an in-depth analysis of memory and nonlinearity trade-offs in neurocomputational systems
Source: Sci Rep. 2023 Jun 29;13:10517. doi: 10.1038/s41598-023-37604-0 (PMC10310772; doi:10.1038/s41598-023-37604-0)
Supplement: Supplementary file 1 — Supplementary Information. [file 41598_2023_37604_MOESM1_ESM.pdf]

Supplementary Material

0.1 Tables

**parameter**	**value**	**description**
V_{min}	0 mV	maximum of the membrane potential distribution
V_{max}	20 mV	minimum of the membrane potential distribution
τ_{m}	20 ms	membrane time constant
C_{m}	1 pF	membrane capacitance
E_{L}	0 mV	resting membrane potential
d	1.5 ms	synaptic delay
V_{th}	20 mV	threshold potential
V_{reset}	10 mV	reset potential
τ_{ref}	2 ms	refractory period
N	1250	network size
N_{exc}	1000	number of excitatory neurons
N_{inh}	250	number of inhibitory neurons
C_{exc}	100	number of incoming excitatory synapses
C_{inh}	25	number of incoming inhibitory synapses
g	5	ratio of inhibitory to excitatory weight
w_{exc}	0.2 pA	excitatory synaptic weight
s_{inh}	$-gw_{\text{exc}} = -1$ pA	inhibitory synaptic weight
ν_{noise}	4000 spk/sec	rate of background noise

Table 1: Parameters for balanced random network

0.2 Figures

The lower half of Figure 1 shows the capacity heat maps for a fixed γ value of 1. We have thus removed all encoder capacities and their delayed versions, resulting in a lower capacity bound. The fact that there are no differences between the results of the different transformation functions tells us that the networks do not compute target functions y_l with an encoder capacity $C^{enc}(y_l) > 0$ better than the encoder does.

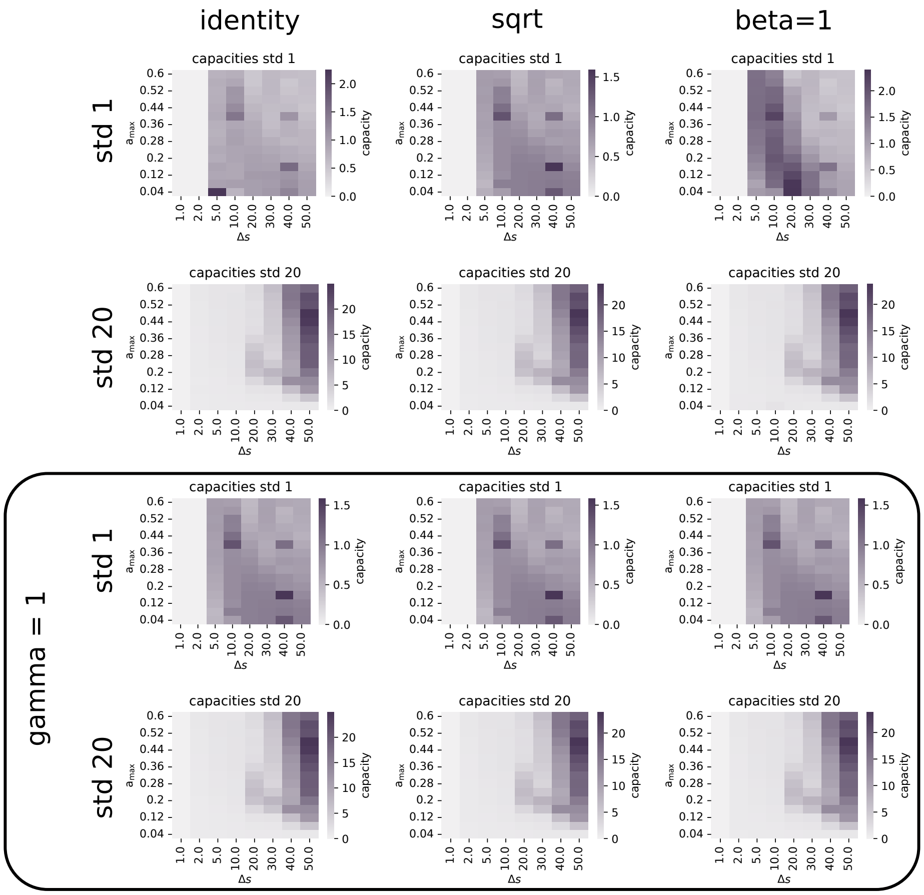

Figure 1: BRN results, spatial encoding, removed remembered encoder capacities

0.3 Details on removing the nonlinear encoder effects

First, we take a closer look at the reconstructions of the target signal and the squared correlation coefficient, which forms the basis for the capacity evaluation. The reconstructed function z_l is a weighted sum of y_l and an uncorrelated noise signal b_l:

$$z_l = \alpha_l \cdot y_l + (1 - \alpha_l) \cdot b_l \tag{1}$$

where α_l is the relative weight of y_l compared to b_l and is related to the capacity:

$$
\begin{aligned}
C_l &= \frac{\mathrm{cov}(y_l, z_l)^2}{\mathrm{var}(y_l)\cdot \mathrm{var}(z_l)} \\
&= \frac{\alpha_l^2 \mathrm{var}(y_l)^2}{\mathrm{var}(y_l)(\alpha_l^2 \cdot \mathrm{var}(y_l) + (1-\alpha_l)^2 \cdot \mathrm{var}(b_l))} \\
&= \frac{\mathrm{var}(y_l)}{\mathrm{var}(y_l) + \mathrm{var}(b_l)\frac{(1-\alpha_l)^2}{\alpha_l^2}} \\
&= \frac{1}{1 + \frac{\mathrm{var}(b_l)}{\mathrm{var}(y_l)}\frac{(1-\alpha_l)^2}{\alpha_l^2}}
\end{aligned}
\tag{2}
$$

Therefore, the relationship between capacity C_l and α_l is nonlinear and depends on the ratio between the variances of b_l and y_l, as Equation 2 and also Figure 2A show. Note that this ratio can be different for each target function.

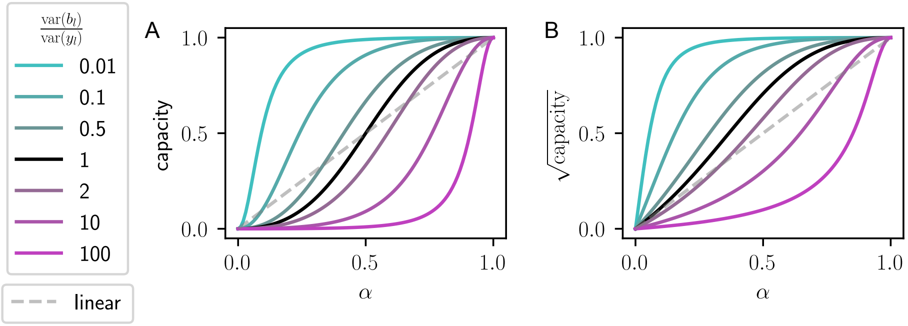

Figure 2: capacity transformation

Following Equation 2 we can define the function f_T that calculates α_l based on the capacity C_l and the ratio of variances β_l of b_l and y_l:

$$
f_T(C_l, \beta_l) = \alpha_l = \frac{1}{\sqrt{\frac{\frac{1}{C_l}-1}{\beta_l}} + 1} \tag{3}
$$

To remove the encoder effects, we first calculate the capacity of the encoder output. We use the resulting capacity profile together with the capacities of the overall system to calculate the effective linear memory that the main system introduces in addition to the encoder memory. To do this, we subtract the encoder memory M^{enc} from the combined memory M^{comb} for each delay i (main Figure 6 B-E):

$$M_i^{\text{sys}} = M_i^{\text{comb}} - M_i^{\text{enc}} \tag{4}$$

M_i is the capacity with input u delayed by i steps as target function.

$$M_i = f_T(C[u(k-i)], \beta_{u_i}) \tag{5}$$

The transformation f_T allows us to obtain meaningful results when we add, subtract or divide the M values under the assumption that we know β_{u_i}.

Based on the system and encoder memory values, we calculate the memory ratio γ for all delays, i.e., the fraction of the encoder input that the system can memorize after a delay i:

$$\gamma_i = \frac{M_i^{\text{sys}}}{M_0^{\text{enc}}} \tag{6}$$

Using these memory ratios, we compute the remembered encoder capacities C^{rem}, i.e., how much a capacity value for a target function can be based on remembering a previous target function that is already computed by the encoder:

$$C^{\text{rem}}(y_n) = f_T^{-1}(f_T(C_0^{\text{enc}}(y_m), \beta_m) \cdot \gamma_i, \beta_m) \tag{7}$$

where $C^{\text{rem}}(y_n)$ is the remembered capacity for the target function y_n, which corresponds to the target function y_m delayed by i steps. These remembered capacities are the result of the linear memory of the system and the nonlinearity and memory of the encoder, and therefore we must subtract them from the capacities of the combined system to obtain the effective capacity of the main system for all objective functions y_l:

$$C^{\text{sys}}(y_l) = f_T^{-1}\left(f_T(C^{\text{comb}}(y_l), \beta_l) - f_T(max(C^{\text{enc}}(y_l), max(C^{rem}(y_l)), \beta_l)\right) \tag{8}$$

where C^{comb} is the measured capacity of the combined system including the encoder and the main system. With this method we can not get information about the precise functions the system can compute as with a linearly encoded input, because the system does not compute the function $F_{\text{sys}}(u)$, but the function $F_{\text{sys}}(F_{\text{enc}}(u))$. Therefore a system capacity $C^{\text{sys}}(y_l) > 0$ does not mean that the system computes the specific target function y_l with the degree d_{y_l}. However, it tells us that the system computes a function which goes beyond

remembering the input signal.

The problem with the transformation f_T is that we do not know the variance ratio β and therefore cannot remove the exact encoder effects. However, we test different ways to approximate f_T. The simplest approximation is to use the identity function and not transform the capacities before calculating γ and subtracting the encoder values. Other possibilities are to take the square root of the capacities to obtain the correlation coefficient instead of its squared value or to set β to a fixed value (e.g. 1) for each target function. To obtain a lower bound for the capacities, we can set γ to 1 for all linear capacities $C[u(k-i)] > 1$. This leads to a complete subtraction of all encoder capacities and their delayed versions and thus to a lower limit for the capacity.
